# Kaposi’s sarcoma associated with chronic myeloid leukemia and imatinib mesylate therapy

**DOI:** 10.1002/ccr3.5919

**Published:** 2022-06-05

**Authors:** Matteo D’Addona, Luca Pezzullo, Valentina Giudice, Bianca Serio, Carlo Baldi, Pio Zeppa, Carmine Selleri

**Affiliations:** ^1^ Hematology and Transplant Center University Hospital “San Giovanni di Dio e Ruggi d’Aragona” Salerno Italy; ^2^ 19028 Department of Medicine, Surgery, and Dentistry University of Salerno Baronissi Italy; ^3^ Anatomy Pathology University Hospital “San Giovanni di Dio e Ruggi d’Aragona” Salerno Italy

**Keywords:** BCR‐ABL inhibitor, chronic myeloid leukemia, Kaposi's sarcoma

## Abstract

Kaposi's sarcoma is associated with immunosuppression and human herpesvirus 8 infection, while rarely described in myeloid malignancies. Here, we illustrate a rare case of chronic myeloid leukemia treated with imatinib, a tyrosine kinase inhibitor, who developed a human herpesvirus 8‐related Kaposi's sarcoma.

## Graphical abstract

Human herpesvirus 8‐related Kaposi's sarcoma development is a rare complication in chronic myeloid leukemia patient under imatinib mesylate treatment likely, associated with its immunosuppressive activity.

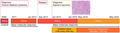

## 1 INTRODUCTION

Kaposi's sarcoma (KS) is an angioproliferative disorder of the skin with five known clinical variants: classical type, prevalent in older Caucasian men with a benign course; African endemic variant, afflicting young men aged 25–40 years; iatrogenic type, associated with immunosuppression (including post‐transplantation and during chemotherapy); endemic variant, related to human immunodeficiency virus (HIV) infection; and novel non‐epidemic form, frequent in HIV seronegative men without other causes of immunodeficiency.^1^ KS is characterized by an uncontrolled proliferation of endothelial or progenitor cells frequently infected by human herpesvirus 8 (HHV8).^1^, ^2^ This γ‐herpesvirus has a seroprevalence of 1%–2% in blood donors, and its genome approximately encodes for 87 open reading frames, including homologous of human interleukin‐6 and interleukin‐8 receptor.^2^ HHV8 has been also associated with the development of B‐cell lymphoproliferative disorders, such as multicentric Castleman disease, multiple myeloma, or primary effusion lymphoma.^3^

Several drugs are related to KS lesion exacerbation, including highly active antiretroviral therapy for HIV infection treatment, corticosteroids, or rituximab, an anti‐CD20 monoclonal antibody used for CD20^+^ B‐cell lymphoma therapy.^2^, ^4^ Association of HHV8^+^ KS and myeloid malignancies are rare and anecdotical reported, especially in patients with chronic myeloid leukemia (CML) treated with specific tyrosine kinase inhibitors, such as imatinib mesylate.^5^ CML, a clonal myeloid hematological malignancy, is characterized by a common reciprocal chromosomal translocation t(9;22)(q34;q11), the so‐called “Philadelphia chromosome,” resulting in the BCR‐ABL1 fusion protein, and myelopoiesis is impaired with immature neutrophils in peripheral blood and bone marrow.^6^

In this report, we present the second‐ever reported case of a patient with CML who developed HHV8^+^ KS under imatinib mesylate treatment.

## 2 CASE REPORT

**FIGURE 1:**
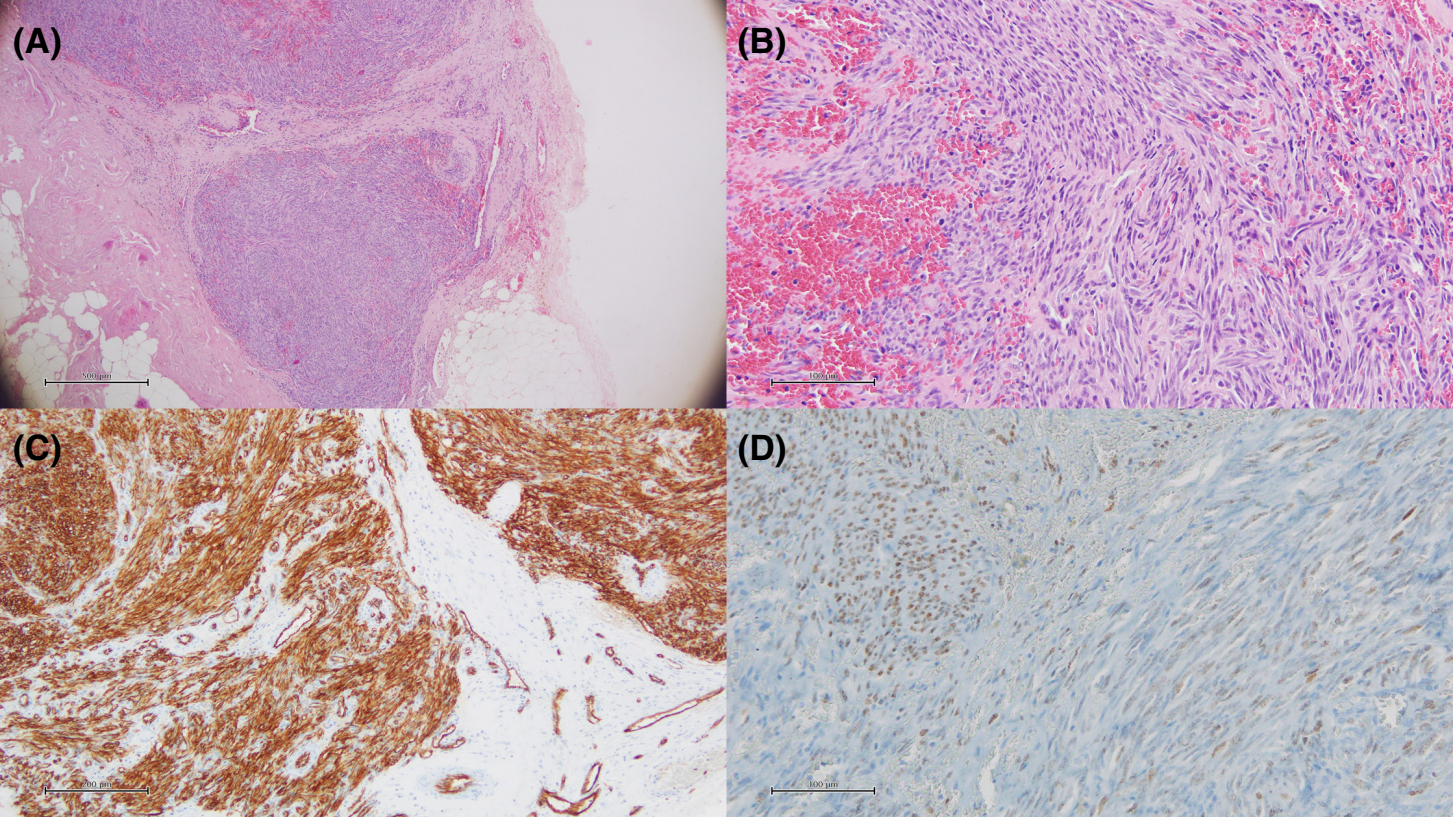
Lymph node biopsy immunohistochemistry. Spindle cells with vascular slits and vascular structures and extravasated erythrocytes are shown at 4x (A) and 20x (B) magnification by optical microscopy using hematoxylin and eosin staining. Vascular structures are identified by CD34 staining (C) and the presence of human herpesvirus 8 (HHV8) within cell nuclei is confirmed by immunostaining using an anti‐C‐terminus of the latent nuclear antigen‐1 molecule of HHV8 antibody (D)

A 70‐year‐old Caucasian male with a medical history of surgery for hemorrhagic gastroduodenal ulcer received a diagnosis of CML with the presence of BCR‐ABL fusion protein in 2009 outside our institution. He first received imatinib mesylate at 400 mg daily until 2010 when treatment was discontinued because of acute renal failure. In 2011, imatinib was reintroduced at 300 mg daily achieving a major molecular response, and the patient was admitted for observation at the Hematology and Transplant Center, University Hospital “San Giovanni di Dio e Ruggi d’Aragona,” Salerno, Italy. In January 2013, red macules appeared to the right lower limb, and skin biopsy was performed showing an inflammatory purpura and fine‐needle aspiration of a right inguinal lymph node displayed reactive hyperplasia. Imatinib was stopped and a Positron Emission Tomography–Computed Tomography (PET/CT) scan was carried out displaying a high F‐18 fluorodeoxyglucose (FDG) radiotracer uptake in right inguinal lymph nodes. Because of disease relapse, imatinib was reintroduced at 300 mg daily in September 2013, achieving a major molecular response in January 2014; however, right iliac, obturator, and inguinal lymph nodes showed persistence of high FDG uptake by PET/CT scan, and lymph node biopsy displayed an infiltrate of KS cells positive for HHV8, CD34, and vimentin, while negative for HIV (Figure 1). A diagnosis of HHV8^+^ KS was made and interferon‐alpha at 3 MU every other day was started. After one year of therapy, PET/CT scan re‐evaluation showed a significant reduction of FDG lymph nodal uptake, and in January 2016, clinical conditions worsened with anemia, increased HHV8 replication activity (copy number, 16,273 copies/ml), and augmented FDG uptake by PET/CT scan while still in major molecular response. In May 2016, the patient started liposomal anthracycline at a cumulative maximal dose of 1260 mg/m^2^ for a total dose of 2142 mg and reduced imatinib mesylate to 200 mg daily. After two months of therapy, PET/CT scan showed a 50% reduction in FDG uptake and size of lesions, and after nine cycles of liposomal anthracycline, HHV8 replication activity was also decreased (from 16,273 to 3232 copies/ml) until complete negativization in June 2021. At the time of writing, the patient is alive and is at the 49th cycle of liposomal anthracycline at 35 mg/m^2^ cumulative dose (total dose, 1,675 mg) without skin lesions. He is also monitored for anthracycline‐related cardiotoxicity with a 58% left ventricular ejection fraction by ultrasound assessment. Flow cytometry immunophenotyping from peripheral blood samples displayed normal frequency of CD19^+^ B lymphocytes (2%), while CD8^+^ T cells were slightly increased compared with CD4^+^ T cells (54% vs. 30%). The patient is still receiving imatinib mesylate at 200 mg daily, maintaining a deep molecular response with an overall survival of 13 years.

## 3 DISCUSSION

KS is a rare angioproliferative disorder frequently occurring in immunosuppressed subjects, such as HIV infected or transplanted patients,^1^, ^2^, ^3^ while cases arising from CML treated with BCR‐ABL inhibitors, like imatinib mesylate, are only anectodical.^5^ Here, we present the second‐ever reported HHV8^+^ KS case in a CML patient under imatinib mesylate treatment who was successfully treated with liposomal anthracycline while maintaining for CML a deep molecular response with low dose imatinib mesylate (200 mg daily). The previous reported case, a 61‐year‐old Caucasian male, showed a KS arising after one year of treatment with 400 mg daily of imatinib mesylate in CML with the presence of BCR‐ABL p210‐b3a2 fusion protein. Our patient developed KS after five years of imatinib at lower dosage (300 mg daily) compared with the previously reported case who was treated with local skin lesion surgical removal resulting in continuous recurrence even after three years from diagnosis.^5^ Conversely, we did not option for surgery, while we decided to a pharmacological treatment, and IFN‐α was chosen as first‐line therapy. However, our patient failed to sustain a complete KS remission under IFN‐α, as usually used alone for treatment of classical KS and HIV‐related variant with normal CD4^+^ T cell count, and a sustained response is observed in around 46% of cases, especially those subjects without visceral involvement.^7^ Our patient displayed the presence of extended inguinal lymph nodal involvement by PET/CT scan that might explain this transient response to IFN‐α therapy.

Before imatinib introduction, IFN‐α based regimens were the main treatment option in CML, and the combination of imatinib and IFN‐α induces a more rapid complete cytogenetic response at six months of therapy and a sustained treatment‐free response compared with imatinib alone, likely because of synergistic effects of these two drugs on immune responses and leukemic cell growth.^8^, ^9^ IFN‐α shows *in vitro* anti‐proliferative effects on CML progenitors by targeting cell cycle progression and pro‐apoptotic proteins and by promoting Natural Killer cell cytolytic activity against leukemic cells. Moreover, IFN‐α inhibits KS cell growth through downregulation of transcription and basic fibroblast growth factors, and by interfering with HHV8 viral life cycle.^8^, ^9^

Imatinib, the first‐in‐class tyrosine kinase inhibitor, mainly blocks BCR‐ABL1 activity and interferes with other tyrosine kinase downstream signaling, such as c‐kit and platelet‐derived growth factor receptor, that are also involved in KS progression. In HIV‐related KS and the reported KS arising from CML, lesion regression rate is low (around 40%) and recurrence is observed even under full dose imatinib treatment.^5^, ^10^ Imatinib exerts immunosuppressive functions by reducing the ability of dendritic cells in stimulating T cells, by inducing T cell anergy, by decreasing CD8^+^ T cell‐mediated responses against viruses likely by altering type I IFN‐mediated responses.^11^ These immunosuppressive actions might be related to uncontrolled HHV8 viral replication and KS development in rare cases. These pleiotropic and synergistic effects of imatinib and IFN‐α might be related to the transient clinical response observed in our patient who instead benefit of liposomal anthracycline treatment, the most frequently administered chemotherapeutic agent in classical KS.^12^

In conclusion, HHV8‐related iatrogenic KS type is more frequently associated with immunosuppression after solid organ or hematopoietic stem cell transplantation and during chemotherapy; however, KS might rarely occur also under imatinib mesylate, a BCR‐ABL1 tyrosine kinase inhibitor, used for treatment of CML. Imatinib reduces CD8+ cytotoxic T cell and type I IFN‐mediated responses, thus decreasing antiviral and immunosurveillance functions of the immune system. We also encourage the use of standard chemotherapy for KS treatment in association with imatinib mesylate for CML control instead of IFN‐α, to minimize immunosuppressive synergistic effects of imatinib and IFN‐α and favor KS progression.

## AUTHOR CONTRIBUTIONS

V.G. and C.S. involved in conceptualization; M.D.A, L.P., V.G., and C.B. involved in clinical data; V.G. and M.D.A. involved in data curation and writing—original draft preparation; C.S. and P.Z. involved in writing—review and editing. All authors have read and agreed to the published version of the manuscript.

## CONFLICTS OF INTEREST

The authors declare no conflict of interest.

## CONSENT

Written informed consent was obtained from the patient to publish this report in accordance with the journal's patient consent policy.

## Data Availability

The data that support the findings of this study are available upon request from the corresponding author. The data are not publicly available due to privacy restrictions.
